# A New Filtering and Smoothing Algorithm for Railway Track Surveying Based on Landmark and IMU/Odometer

**DOI:** 10.3390/s17061438

**Published:** 2017-06-19

**Authors:** Qingan Jiang, Wenqi Wu, Mingming Jiang, Yun Li

**Affiliations:** Department of Automatic Control, College of Mechatronics and Automation, National University of Defense Technology, Changsha 410073, China; jqa1987@nudt.edu.cn (Q.J.); uninavi@163.com (M.J.); liyun2009@nudt.edu.cn (Y.L.)

**Keywords:** railway track surveying, filtering and smoothing, IMU, total station, odometer, covariance analysis

## Abstract

High-accuracy railway track surveying is essential for railway construction and maintenance. The traditional approaches based on total station equipment are not efficient enough since high precision surveying frequently needs static measurements. This paper proposes a new filtering and smoothing algorithm based on the IMU/odometer and landmarks integration for the railway track surveying. In order to overcome the difficulty of estimating too many error parameters with too few landmark observations, a new model with completely observable error states is established by combining error terms of the system. Based on covariance analysis, the analytical relationship between the railway track surveying accuracy requirements and equivalent gyro drifts including bias instability and random walk noise are established. Experiment results show that the accuracy of the new filtering and smoothing algorithm for railway track surveying can reach 1 mm (1σ) when using a Ring Laser Gyroscope (RLG)-based Inertial Measurement Unit (IMU) with gyro bias instability of 0.03°/h and random walk noise of 0.005${}^{\circ}/\sqrt{h}$ while control points of the track control network (CPIII) position observations are provided by the optical total station in about every 60 m interval. The proposed approach can satisfy at the same time the demands of high accuracy and work efficiency for railway track surveying.

## 1. Introduction

Railway tracks will drift away from their designed position due to external factors, which could cause track deformation and irregularities [1,2]. The railway track geometry parameter is one of the important performance indexes of track smoothness for monitoring the track deformation and guiding the maintenance of railway lines. The track geometry parameters including the inner geometry parameters and the outer geometry parameters have different accuracy requirements concerning the track course smoothness and the absolute position of the track [1,3]. The inner geometry parameters are quantified by the relative accuracy and the outer geometry parameters by the absolute accuracy [1,3]. Both of them must be guaranteed to ensure the safety operation. High-speed railway track structures require high smoothness. With the rapid development of high-speed railways, railway track surveying methods of high performance are more and more important to guarantee the trains’ operational safety and stability. A perfect track surveying method should provide high relative or absolute accuracy effectively without interfering with regular train traffic [1].

At present, the common track surveying methods can be generally divided into two categories, namely static measurement and dynamic measurement. The traditional track surveying techniques include manual measuring devices or Track Recording Vehicles (TRVs) [4]. The track surveying trolley is a new segment in the category. As a kind of quasi-static track surveying equipment the trolley plays an important role in railway track surveying. The geometry parameters of the track are measured by the measuring equipment mounted on the trolley, such as inclinators, accelerometers, gyroscopes, odometers, laser scanners, total stations, Global Positioning System (GPS) receivers and so on, when the trolley moves on the track driven by people or a motor.

Track surveying trolleys have claimed areas of application where they are superior to other traditional techniques. Measurements during the construction stage and measurements of shorter stretches of track are some areas where the trolleys outperform TRVs due to their light weight and flexible characteristics [4]. Manual devices used for spot assessment have been quickly superseded by trolleys due to time efficiency [4].

Nowadays the railway track surveying trolley is widely used in railway construction and maintenance projects. The GRP1000 track surveying trolley provided by the Swiss company Amberg Technologies (Regensdorf, Switzerland) and the GEDO CE one provided by Sinning (Wiesentheid, Germany) are widely deployed in China [2]. These kinds of trolleys equipped with optical total stations can measure the track position with millimeter or even sub-millimeter accuracy in stop-and-go mode [1,2]. Another representative track surveying trolley, namely the Swiss trolley [1,4], was developed by the Institute of Geodesy and Photogrammetry at ETH Zurich in collaboration with a number of others. Glaus [1] describes this trolley thoroughly and comprehensively in his thesis. The basic configuration of the Swiss trolley contains an inclinator, track gauge measuring system and odometer, enabling the assessment of the cant, gradient, track gauge, chainage and twist parameters. The positioning configuration equipped with Real Time Kinematic GPS (GPS-RTK) and optical total stations allows for absolute position fixing. Glaus designed two Kalman filter models and a smoother to fuse the data measured by GPS and other sensors to improve the measurement precision.

Although the surveying trolley based on an optical total station can satisfy the precision requirement and has achieved the most extensive application in railway track surveying, there are still some shortcomings. The high precision measurement by total station requests the trolley to be operated in static measurement mode [1,2]. The distance between two position measurement points is short (10 m interval or less) which makes it not efficient enough for track surveying [2,4].

A method based on the integration of Differential GPS (DGPS) with an Inertial Navigation System (INS) is a new measuring technique approach for railway track surveying in order to overcome the deficiencies of the total station method. Applanix Corporation [5] developed the POS/TG system which consists of an IMU, a GPS receiver, a Distance Measurement Indicator and an Optical Gauge Measurement system, for dynamic inspection train. It has been successfully employed by the Austrian Federal railways. Luck [6,7] obtained relative accuracy in the millimeter range for track surveying by integration of DGPS and INS. He established a forward and backward Kalman smoother to reduce the variances of the position solution, and pointed out some limiting factors such as synchronization and lever arm between different sensors, must be considered under the prerequisite for high accuracy surveying in the millimeter range.

Niu and Chen [2,8] at Wuhan University used the Global Navigation Satellite System and Inertial Navigation System (GNSS/INS) integrated technique for railway track irregularity surveying, and achieved high relative accuracy of 1 mm and absolute accuracy of several centimeters in the kinematic mode. They developed a modified integration algorithm to compensate the drift of inertial sensors, and implemented the non-holonomic constraint and zero velocity updates in the Kalman filter to improve the surveying accuracy.

However, a critical issue is that the GPS signal may become obstructed by tunnels, bridges and other obstacles. This affects the GPS solutions negatively. In order to carry out the surveying task with poor GPS receptions or with GPS outages, Niu used the total station instead of GPS in combination with INS for the absolute position measurement and proposed the measure scheme briefly, but their solution still needs short interval position observations by a total station and they have not provided details of an algorithm. Nassar, Liu, and EI-Sheimy [9,10] took advantage of a fixed-interval smoother to increase the position accuracy during short interval GPS outages for INS/GPS applications, but it cannot satisfy the demands of track surveying during long duration GPS outages. In this case other relative or absolute sensors with complementary properties are introduced to depress the error drift, such as odometers, Doppler laser radars, cameras, or landmarks. Wu [11,12] proposed a versatile strategy for land vehicle navigation using IMU/odometer integration. A self-calibration method and an odometer-aided IMU in-motion alignment algorithm were devised in his work. De Cecco [13] presented a sensor-fusion algorithm for navigation systems suitable for autonomous guided vehicles that uses two measurement systems: an odometric one and an inertial one. Vettori and Malvezzi [14] described a pose estimation algorithm using INS/odometer integration to increase the accuracy of the odometric estimation, especially in critical adhesion conditions. Liu and Nassar [15] obtained successful utility in pipeline surveying by INS with the aid of an odometer and control points.

Traditional integrated navigation systems based on IMUs, odometers, and landmarks have different integration solutions, such as: INS/landmark integration, INS/odometer/landmark integration, and IMU/odometer/landmark integration which means the integrated landmark and dead reckoning based on gyros and odometer in this paper. They have been widely used in land vehicle navigation and some other applications [11,12,15], but railway track surveying has its own characteristics. For example, it needs high measurement accuracy in the millimeter range [3]. The railway track is so level and straight that the trolley maneuvers are rather weak when moving on the track at low speed. Many error parameters are then coupled together with others, such as, the orientation error is coupled with the equivalent east gyro bias, and the level errors are coupled with equivalent horizontal accelerometer biases. Furthermore, for the surveying tasks without GPS, the observations of control points are few in order to improve the efficiency of measurement. It is difficult to estimate so many error parameters with so few observations under weak maneuver conditions.

This paper focuses on the problem of railway track surveying based on the IMU/odometer/landmark integration to overcome the problems above. After completing initial alignment, we make an attempt to use the attitude measured by a gyro assembly and the distance measured by an odometer for dead reckoning integrated with landmarks about every 60 m interval. This is the distance between two CPIII control points. The absolute position of the landmark is measured by a high precision total station. In order to overcome the difficulty of estimating too many error parameters with too few landmark observations, a new Kalman filter model with completely observable error states is established by combining the error terms of the system. In this way, the equivalent gyro biases can be established and compensated effectively. Moreover, a fixed-interval smoothing algorithm is devised to increase the position accuracy between two control points. Based on covariance analysis, the surveying precision of the proposed algorithm is presented, the analytical relationship between the surveying accuracy and equivalent gyro drifts including bias instability and random walk noise are established. Simulation and real experimental results are also presented compared with traditional algorithms.

This paper is organized as follows: Section 2 describes the overview of surveying system and the new algorithm. Section 3 describes the error model of IMU/odometer based dead reckoning systems. Section 4 presents the new Kalman filtering and smoothing algorithm. Section 5 presents theoretical analyses of the performance for the new method. Section 6 reports the experimental results of railway track surveying. Section 7 concludes this paper.

## 2. Overview of the Surveying System and Algorithm

**Figure 1:**
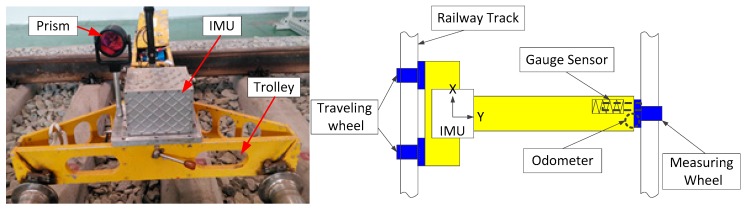
The proposed surveying system and its configuration diagram.

As illustrated in Figure 1, the surveying system is equipped with a T-type trolley, an odometer, a track gauge sensor, a high precision prism and a navigation grade IMU. The IMU consists of three high accuracy ring laser gyros with bias instability of 0.03°/h and random walk noise of 0.005${}^{\circ}/\sqrt{h}$ and three high stability quartz accelerometers (50 μg, 10 μg/$\sqrt{\mathrm{Hz}}$). The prism mounted on the trolley is used to measure the absolute position by the high precision Leica total station (0.6 mm, 0.5”) based on the CPIII control points. In addition, we can also mount the total station on the trolley instead of the prism to reduce the leveling time of the total station.

**Figure 2:**
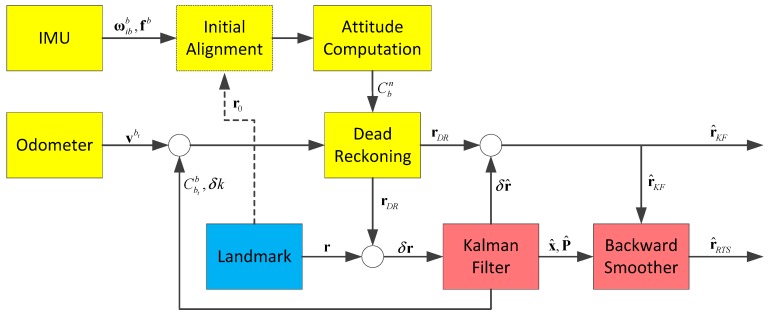
Block diagram of the filtering and smoothing algorithm.

The cost of the IMU used here is about $70,000. It is much more expensive than other sensors in the system except for high precision total station which is essential for high accuracy absolute position measurement. Therefore, it is significant for cost reduction to reduce the performance demand of initial sensors and not use accelerometers. Figure 2 illustrates an overview of the data processing procedure of the Kalman filtering and smoothing algorithm.

The system makes use of the attitude measured by the gyro assembly and the velocity or distance increment measured by the odometer for dead reckoning. When the system comes across a landmark, a position observation updates, and the Kalman filtering and smoothing algorithm is executed to output the optimized position measurements of the interval. The initial attitudes of the system can be provided by initial alignment of the IMU or some other methods.

## 3. Error Model of Dead Reckoning Based on IMU/Odometer

### 3.1. Attitude Error Equation

The attitude error equation may be expressed as shown in Equation (1) [16]:

(1) $${\dot{\phi }}^{n}=-\omega_{in}^{n}\times \phi^{n}+\delta \omega_{in}^{n}-C_{b}^{n}\delta \omega_{ib}^{b}$$

where *i*-frame represents the inertial frame. *n*-frame is the local level frame (North-East-Down) used as the navigation frame. *b*-frame is the body frame of the IMU (Forward-Right-Down). $\phi^{n}={\left[\begin{array}{ccc} \phi_{N} & \phi_{E} & \phi_{D} \end{array}\right]}^{T}$ represents the vector of attitude errors about the north, east and down axes of the navigation frame. $\omega_{in}^{n}$ represents the turn rate of the navigation frame with respect to the inertial frame expressed in the *n*-frame. it can be obtained by summing the Earth’s rotation rate with respect to the inertial frame and the turn rate of the navigation frame with respect to the Earth as: $\omega_{in}^{n}=\omega_{ie}^{n}+\omega_{en}^{n}$ · $\delta \omega_{in}^{n}$ represents the errors of the navigation frame rate. $\delta \omega_{ib}^{b}$ represents the drift errors of gyroscopes. $C_{b}^{n}$ represents the direction cosine matrix.

Considering that the track surveying trolley is pushed forward manually at walking speed and the surveying distance is a quite short interval, we can ignore the turn rate of the navigation frame with respect to the Earth. The attitude error equation can be rearranging as shown by Equation (2):

(2) $$\left[\begin{array}{c} {\dot{\phi }}_{N}\\ {\dot{\phi }}_{E}\\ {\dot{\phi }}_{D} \end{array}\right]\approx \left[\begin{array}{ccc} 0 & \omega_{ieD} & 0\\ -\omega_{ieD} & 0 & \omega_{ieN}\\ 0 & -\omega_{ieN} & 0 \end{array}\right]\left[\begin{array}{c} \phi_{N}\\ \phi_{E}\\ \phi_{D} \end{array}\right]-C_{b}^{n}\left[\begin{array}{c} \delta \omega_{x}^{b}\\ \delta \omega_{y}^{b}\\ \delta \omega_{z}^{b} \end{array}\right]$$

In addition, since the high speed railway requires a level and straight railway track, the radius of curvature of the track is usually very large and the track gradient is very small. Therefore the level attitudes are small angles for the surveying trolley and the direction cosine matrix here can be written in component form as shown by Equation (3) [16,17]:

(3) $$C_{b}^{n}\approx \left[\begin{array}{ccc} \cos\varphi & -\sin\varphi & \theta \cos\varphi +\gamma \sin\varphi \\ \sin\varphi & \cos\varphi & -\gamma \cos\varphi +\theta \sin\varphi \\ -\theta & \gamma & 1 \end{array}\right]$$

where γ, θ, φ represent the Euler rotation angles about the roll pitch and yaw axes, respectively.

The level errors $\phi_{N}$ and $\phi_{E}$ can be equivalently converted into error angles rotating around the longitudinal direction and lateral direction of the trolley, denoted by $\phi_{roll}$ and $\phi_{pitch}$, respectively, by rotating an angle φ around the vertical axis. That is:

(4) $$\left[\begin{array}{c} \phi_{roll}\\ \phi_{pitch}\\ \phi_{D} \end{array}\right]=\left[\begin{array}{ccc} \cos\varphi & \sin\varphi & 0\\ -\sin\varphi & \cos\varphi & 0\\ 0 & 0 & 1 \end{array}\right]\left[\begin{array}{c} \phi_{N}\\ \phi_{E}\\ \phi_{D} \end{array}\right],\;\left[\begin{array}{c} \phi_{N}\\ \phi_{E}\\ \phi_{D} \end{array}\right]=\left[\begin{array}{ccc} \cos\varphi & -\sin\varphi & 0\\ \sin\varphi & \cos\varphi & 0\\ 0 & 0 & 1 \end{array}\right]\left[\begin{array}{c} \phi_{roll}\\ \phi_{pitch}\\ \phi_{D} \end{array}\right]$$

Differentiating Equation (4), substituting Equation (2) and rearranging yields:

(5) $$\left[\begin{array}{c} {\dot{\phi }}_{roll}\\ {\dot{\phi }}_{pitch}\\ {\dot{\phi }}_{D} \end{array}\right]=\left[\begin{array}{ccc} 0 & \omega_{ieD}+\dot{\varphi } & \omega_{ieN}\sin\varphi \\ -\omega_{ieD}-\dot{\varphi } & 0 & \omega_{ieN}\cos\varphi \\ -\omega_{ieN}\sin\varphi & -\omega_{ieN}\cos\varphi & 0 \end{array}\right]\left[\begin{array}{c} \phi_{roll}\\ \phi_{pitch}\\ \phi_{D} \end{array}\right]-\left[\begin{array}{ccc} 1 & 0 & \theta \\ 0 & 1 & -\gamma \\ -\theta & \gamma & 1 \end{array}\right]\left[\begin{array}{c} \delta \omega_{x}^{b}\\ \delta \omega_{y}^{b}\\ \delta \omega_{z}^{b} \end{array}\right]$$

Rearranging Equation (5) gives:

(6) $$\left[\begin{array}{c} {\dot{\phi }}_{roll}\\ {\dot{\phi }}_{pitch}\\ {\dot{\phi }}_{D} \end{array}\right]=-\left[\begin{array}{c} \delta \omega_{x}^{b}-\omega_{ieD}\phi_{pitch}-\dot{\varphi }\phi_{pitch}-\omega_{ieN}\phi_{D}\sin\varphi \\ \delta \omega_{y}^{b}+\omega_{ieD}\phi_{roll}+\dot{\varphi }\phi_{roll}-\omega_{ieN}\phi_{D}\cos\varphi \\ \delta \omega_{z}^{b}+\omega_{ieN}\phi_{roll}\sin\varphi +\omega_{ieN}\phi_{pitch}\cos\varphi \end{array}\right]=-\left[\begin{array}{c} \delta {\bar{\omega }}_{x}^{b}\\ \delta {\bar{\omega }}_{y}^{b}\\ \delta {\bar{\omega }}_{z}^{b} \end{array}\right]+\left[\begin{array}{c} w_{roll}\\ w_{pitch}\\ w_{D} \end{array}\right]$$

where ${\left[\begin{array}{ccc} \delta {\bar{\omega }}_{x}^{b} & \delta {\bar{\omega }}_{y}^{b} & \delta {\bar{\omega }}_{z}^{b} \end{array}\right]}^{T}$ represents the equivalent bias instability of the gyroscopes. ${\left[\begin{array}{ccc} w_{roll} & w_{pitch} & w_{D} \end{array}\right]}^{T}$ represents the equivalent random walk noise of the gyroscopes.

### 3.2. Position Error Equation

Position errors may be caused by attitude errors, including initial alignment errors and attitude errors caused by gyro drifts, the mounting misalignment between IMU and the trolley, and the scale-factor of the odometer. When using the attitude measured by IMU and the velocity measured by odometer for dead reckoning, the estimated trolley position equation may be written in terms of the errors using Equation (7):

(7) $$\Delta {\dot{\tilde{r}}}^{n}={\tilde{C}}_{b}^{n}C_{b_{t}}^{b}\left[\begin{array}{c} {\tilde{v}}_{odo}\\ 0\\ 0 \end{array}\right]$$

where $b_{t}$-frame is the body frame of trolley, the axes of which align with the forward, right and down directions of the trolley. The estimated position vector is denoted by $\Delta {\tilde{r}}^{n}$ and $\Delta {\tilde{r}}^{n}={\left[\begin{array}{ccc} \Delta {\tilde{r}}_{N} & \Delta {\tilde{r}}_{E} & \Delta {\tilde{r}}_{D} \end{array}\right]}^{T}$. It is the relative position vector of trolley from the last landmark projected in the navigation frame. The estimated transform matrix from the body frame to the navigation frame is expressed as ${\tilde{C}}_{b}^{n}$ with attitude errors. The coordinate transform matrix with mounting misalignments of the IMU with respect to the trolley is denoted by $C_{b_{t}}^{b}$. ${\tilde{v}}_{odo}$ is the velocity of the trolley measured by the odometer. For small angular misalignments, the above direction cosine matrix ${\tilde{C}}_{b}^{n}$ can be written by Equation (8):

(8) $${\tilde{C}}_{b}^{n}=\left[I-\left(\phi^{n}\times \right)\right]C_{b}^{n}$$

The skew symmetric form of attitude errors vector is:

(9) $$\phi^{n}\times =\left[\begin{array}{ccc} 0 & -\phi_{D} & \phi_{E}\\ \phi_{D} & 0 & -\phi_{N}\\ -\phi_{E} & \phi_{N} & 0 \end{array}\right]$$

Similarly, for small angular mounting misalignments, the matrix $C_{b_{t}}^{b}$ can be written as shown by Equation (10):

(10) $$C_{b_{t}}^{b}=\left[\begin{array}{ccc} 1 & \varepsilon_{z} & -\varepsilon_{y}\\ -\varepsilon_{z} & 1 & \varepsilon_{x}\\ \varepsilon_{y} & -\varepsilon_{x} & 1 \end{array}\right]$$

where $\varepsilon_{x}$, $\varepsilon_{y}$ and $\varepsilon_{z}$ are the misalignments of IMU about the forward, right and down axes of the trolley frame.

The estimated velocity measured by odometer may be written as shown in Equation (11):

(11) $$\begin{array}{l} {\tilde{v}}_{odo}=\left(1+\delta k\right)v_{odo}\\ \delta \dot{k}=w_{\delta k} \end{array}$$

where δk is the scale factor error of the odometer. $w_{\delta k}$ is the random noise of the odometer scale factor.

Combining and rearranging Equations (7)–(11) above and ignoring the higher order terms yields the following position error equation:

(12) $$\delta \Delta {\dot{r}}^{n}=\Delta {\dot{\tilde{r}}}^{n}-\Delta {\dot{r}}^{n}\approx \left[\begin{array}{ccc} 0 & \theta & \sin\varphi \\ -\theta & 0 & -\cos\varphi \\ -\sin\varphi & \cos\varphi & 0 \end{array}\right]\left[\begin{array}{c} \phi_{N}\\ \phi_{E}\\ \phi_{D} \end{array}\right]v_{odo}+\left[\begin{array}{ccc} \cos\varphi & -\sin\varphi & \theta \cos\varphi +\gamma \sin\varphi \\ \sin\varphi & \cos\varphi & -\gamma \cos\varphi +\theta \sin\varphi \\ -\theta & \gamma & 1 \end{array}\right]\left[\begin{array}{c} \delta k\\ -\varepsilon_{z}\\ \varepsilon_{y} \end{array}\right]v_{odo}$$

Since the level attitude angles are small angles, the product of level attitude angles (θ, γ) with the level errors ($\phi_{N}$, $\phi_{E}$), the scale factor error (δk) and the misalignment errors ($\varepsilon_{y}$, $\varepsilon_{z}$) can be neglected in a short interval. Hence, the position error equation can be written using Equation (13):

(13) $$\delta \Delta {\dot{r}}^{n}\approx \left[\begin{array}{ccc} 0 & 0 & \sin\varphi \\ 0 & 0 & -\cos\varphi \\ -\sin\varphi & \cos\varphi & 0 \end{array}\right]\left[\begin{array}{c} \phi_{N}\\ \phi_{E}\\ \phi_{D} \end{array}\right]v_{odo}+\left[\begin{array}{c} \cos\varphi \\ \sin\varphi \\ 0 \end{array}\right]\delta kv_{odo}+\left[\begin{array}{c} \sin\varphi \\ -\cos\varphi \\ 0 \end{array}\right]\varepsilon_{z}v_{odo}+\left[\begin{array}{c} 0\\ 0\\ 1 \end{array}\right]\varepsilon_{y}v_{odo}$$

Substituting Equation (4) into Equation (13) and rearranging it yields:

(14) $$\begin{array}{ll} \delta \Delta {\dot{r}}^{n} & \approx \left[\begin{array}{ccc} 0 & 0 & \sin\varphi \\ 0 & 0 & -\cos\varphi \\ 0 & 1 & 0 \end{array}\right]\left[\begin{array}{c} \phi_{roll}\\ \phi_{pitch}\\ \phi_{D} \end{array}\right]v_{odo}+\left[\begin{array}{c} \cos\varphi \\ \sin\varphi \\ 0 \end{array}\right]\delta kv_{odo}+\left[\begin{array}{c} \sin\varphi \\ -\cos\varphi \\ 0 \end{array}\right]\varepsilon_{z}v_{odo}+\left[\begin{array}{c} 0\\ 0\\ 1 \end{array}\right]\varepsilon_{y}v_{odo}\\ & =\left[\begin{array}{ccc} 0 & \sin\varphi & \cos\varphi \\ 0 & -\cos\varphi & \sin\varphi \\ 1 & 0 & 0 \end{array}\right]\left[\begin{array}{c} \phi_{pitch}+\varepsilon_{y}\\ \phi_{D}+\varepsilon_{z}\\ \delta k \end{array}\right]v_{odo}\\ & =\left[\begin{array}{ccc} 0 & \sin\varphi & \cos\varphi \\ 0 & -\cos\varphi & \sin\varphi \\ 1 & 0 & 0 \end{array}\right]\left[\begin{array}{c} {\bar{\phi }}_{pitch}\\ {\bar{\phi }}_{D}\\ \delta k \end{array}\right]v_{odo} \end{array}$$

where ${\left[\begin{array}{cc} {\bar{\phi }}_{pitch} & {\bar{\phi }}_{D} \end{array}\right]}^{T}$ represents the equivalent attitude error angles of the surveying trolley.

## 4. A New Kalman Filtering and Smoothing Algorithm for Railway Track Surveying

### 4.1. New Kalman Filter Designed for Track Surveying

Track surveying trolley maneuvers are rather weak when moving on the track at walking speed. It is difficult to estimate all the system error states with so few landmark position observations. Therefore we do not need establish a Kalman filter equation including all the system errors. As shown in Equation (14), the error angle $\phi_{roll}$ evokes non-significant position errors in the three directions in a short period of time. We can reduce the dimensionality of the Kalman filter by excluding the unobservable state $\phi_{roll}$ and Equation (6) can be transformed as shown in Equation (15):

(15) $$\left[\begin{array}{c} {\dot{\phi }}_{pitch}\\ {\dot{\phi }}_{D} \end{array}\right]\approx -\left[\begin{array}{c} \delta {\bar{\omega }}_{y}^{b}\\ \delta {\bar{\omega }}_{z}^{b} \end{array}\right]+\left[\begin{array}{c} w_{pitch}\\ w_{D} \end{array}\right]$$

Considering the definition of the equivalent attitude error angles in Equation (14), we can get:

(16) $$\left[\begin{array}{c} {\dot{\bar{\phi }}}_{pitch}\\ {\dot{\bar{\phi }}}_{D} \end{array}\right]=\left[\begin{array}{c} {\dot{\phi }}_{pitch}+{\dot{\varepsilon }}_{y}\\ {\dot{\phi }}_{D}+{\dot{\varepsilon }}_{z} \end{array}\right]=\left[\begin{array}{c} {\dot{\phi }}_{pitch}\\ {\dot{\phi }}_{D} \end{array}\right]\approx -\left[\begin{array}{c} \delta {\bar{\omega }}_{y}^{b}\\ \delta {\bar{\omega }}_{z}^{b} \end{array}\right]+\left[\begin{array}{c} w_{pitch}\\ w_{D} \end{array}\right]$$

Since there is no coupling between the vertical and the level channel, we can establish two reduced-order Kalman filters in the vertical and the level channel, respectively. For the vertical channel, the system error model and observation model can be expressed using Equation (17):

(17) $$\begin{array}{l} {\dot{x}}_{v}\left(t\right)=A_{v}x_{v}\left(t\right)+G_{v}w_{v}\left(t\right)\\ z_{v}\left(t\right)=H_{v}x_{v}\left(t\right)+v_{v}\left(t\right) \end{array}$$

where the error state vector of vertical channel $x_{v}\left(t\right)$ can be written as Equation (18):

(18) $$x_{v}\left(t\right)={\left[\begin{array}{ccc} \delta \Delta r_{D} & {\bar{\phi }}_{pitch} & \delta {\bar{\omega }}_{y}^{b} \end{array}\right]}^{T}$$

The system error matrix $A_{v}$ and the noise matrix $G_{v}$ can be expressed in full by Equation (19):

(19) $$A_{v}=\left[\begin{array}{ccc} 0 & v_{odo} & 0\\ 0 & 0 & -1\\ 0 & 0 & 0 \end{array}\right],\;G_{v}=\left[\begin{array}{c} 0\\ 1\\ 0 \end{array}\right]$$

The system random walk noise of vertical channel $w_{v}\left(t\right)∼N\left(0,Q_{v}\left(t\right)\right)$ can be expressed as shown by Equation (20):

$Q_{v}\left(t\right)=\sigma_{w_{pitch}}^{2}$ represents the Power Spectral Density (PSD) of the vertical channel system noise.

(20) $$w_{v}\left(t\right)=w_{pitch}$$

The measurement differences between position provided by the total station and the estimates of these measurements obtained from the dead reckoning system constitute the Kalman filter innovation. For the vertical channel the innovation $z_{v}\left(t\right)=\delta \Delta r_{D}$ and the measurement matrix $H_{v}$ are defined by Equation (21):

(21) $$H_{v}=\left[\begin{array}{ccc} 1 & 0 & 0 \end{array}\right]$$

$\upsilon_{v}\left(t\right)=\upsilon_{r_{D}},\;\upsilon_{v}\left(t\right)∼N\left(0,R_{v}\left(t\right)\right)$ represents the measurement noise of the vertical channel, and $R_{v}\left(t\right)=\sigma_{r}^{2}$ is the PSD of it.

For the level channel, the system error model and observation model can be expressed as indicated in Equation (22):

(22) $$\begin{array}{l} {\dot{x}}_{l}\left(t\right)=A_{l}x_{l}\left(t\right)+G_{l}w_{l}\left(t\right)\\ z_{l}\left(t\right)=H_{l}x_{l}\left(t\right)+v_{l}\left(t\right) \end{array}$$

where:

(23) $$x_{l}\left(t\right)={\left[\begin{array}{ccccc} \delta \Delta r_{N} & \delta \Delta r_{E} & \delta k & {\bar{\phi }}_{D} & \delta {\bar{\omega }}_{z}^{b} \end{array}\right]}^{T}$$

(24) $$A_{l}=\left[\begin{array}{ccccc} 0 & 0 & v_{odo}\cos\varphi & v_{odo}\sin\varphi & 0\\ 0 & 0 & v_{odo}\sin\varphi & -v_{odo}\cos\varphi & 0\\ 0 & 0 & 0 & 0 & 0\\ 0 & 0 & 0 & 0 & -1\\ 0 & 0 & 0 & 0 & 0 \end{array}\right],G_{l}=\left[\begin{array}{cc} 0 & 0\\ 0 & 0\\ 1 & 0\\ 0 & 1\\ 0 & 0 \end{array}\right],H_{l}=\left[\begin{array}{ccccc} 1 & 0 & 0 & 0 & 0\\ 0 & 1 & 0 & 0 & 0 \end{array}\right]$$

(25) $$\begin{array}{l} w_{l}\left(t\right)={\left[\begin{array}{cc} w_{\delta k} & w_{D} \end{array}\right]}^{T},\;w_{l}\left(t\right)∼N\left(0,Q_{l}\left(t\right)\right)\\ Q_{l}\left(t\right)=diag\left(\left[\begin{array}{cc} \sigma_{w_{\delta k}}^{2} & \sigma_{w_{D}}^{2} \end{array}\right]\right)\\ \upsilon_{l}\left(t\right)={\left[\begin{array}{cc} \upsilon_{r_{N}} & \upsilon_{r_{E}} \end{array}\right]}^{T},\;\upsilon_{l}\left(t\right)∼N\left(0,R_{l}\left(t\right)\right)\\ R_{l}\left(t\right)=diag\left(\left[\begin{array}{cc} \sigma_{r}^{2} & \sigma_{r}^{2} \end{array}\right]\right) \end{array}$$

### 4.2. Observability Analysis

In the process of railway track surveying, the change of attitude is slow in short distance intervals and the velocity of the trolley is almost constant. It is therefore feasible to simplify the surveying process as a uniform motion in a straight line, so the Kalman filter established in the last section is a linear time-invariant system and the observability can be analyzed straightforwardly by testing the rank of the observability matrix.

According to the definition, the rank for observability matrixes of the vertical channel and level channel can be expressed using Equation (26):

(26) $$rank\left[\begin{array}{c} H_{v}\\ H_{v}A_{v}\\ H_{v}A_{v}^{2} \end{array}\right]=rank\left[\begin{array}{ccc} 1 & 0 & 0\\ 0 & v_{odo} & 0\\ 0 & 0 & -v_{odo} \end{array}\right]=3\;rank\left[\begin{array}{c} H_{l}\\ H_{l}A_{l}\\ \vdots \\ H_{l}A_{l}^{4} \end{array}\right]=rank\left[\begin{array}{ccccc} 1 & 0 & 0 & 0 & 0\\ 0 & 1 & 0 & 0 & 0\\ 0 & 0 & v_{odo}\cos\varphi & v_{odo}\sin\varphi & 0\\ 0 & 0 & v_{odo}\sin\varphi & -v_{odo}\cos\varphi & 0\\ 0 & 0 & 0 & 0 & v_{odo}\sin\varphi \\ 0 & 0 & 0 & 0 & v_{odo}\cos\varphi \\ 0_{4\times 1} & 0_{4\times 1} & 0_{4\times 1} & 0_{4\times 1} & 0_{4\times 1} \end{array}\right]=5$$

Since both of them have a full rank observability matrix, the error states of the two Kalman filters can be estimated.

### 4.3. Smoothing Algorithm

**Figure 3:**
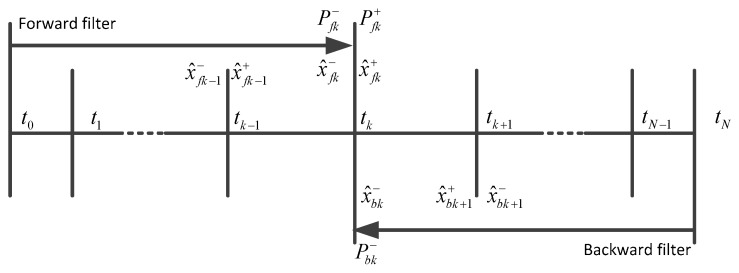
Two-filter smoothing algorithm computational process.

The positions measured by total station are considered as updates for the Kalman filter, and thus, the position errors as well as the covariance information of the integrated IMU/odometer/landmark solution will be very small at the observation points. However, the position errors and their covariance increase with time between two observation points due to the residual system errors. In order to obtain accurate positions during the observation outages, a bridging algorithm must be used for estimating improved positions for these periods. This is a typical fixed interval smoothing problem. The Two-Filter Smoother (TFS) and the Rauch-Tung-Striebel (RTS) smoother are two classical smoothing methods [9,18]. The TFS includes a forward Kalman filter and a backward Kalman filter, and the smoothed estimate of state vector is calculated by combining the forward and the backward filtered solutions. Figure 3 illustrates the computation procedure.

The TFS algorithm in discrete form is shown in Equation (27) [17,18,19]:

(27) $$\begin{array}{l} {\hat{x}}_{k}=P_{k}\left[{\left(P_{fk}^{+}\right)}^{-1}{\hat{x}}_{fk}^{+}+{\left(P_{bk}^{-}\right)}^{-1}{\hat{x}}_{bk}^{-}\right]\\ P_{k}={\left[{\left(P_{fk}^{+}\right)}^{-1}+{\left(P_{bk}^{-}\right)}^{-1}\right]}^{-1} \end{array}$$

where ${\hat{x}}_{k}$ is the optimal smoothed estimate of state vector at time epoch k. $P_{k}$ is the error state covariance matrix of the smoother. ${\hat{x}}_{fk}^{+}$ and $P_{fk}^{+}$ represent the updated estimate of state vector and its corresponding covariance matrix of the forward filter at epoch k. ${\hat{x}}_{bk}^{-}$ and $P_{bk}^{-}$ represent the optimal predicted estimate of state vector and its corresponding covariance matrix of the backward filter at epoch k.

**Figure 4:**
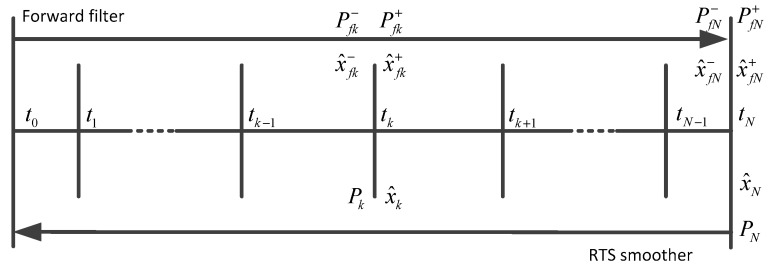
RTS smoothing algorithm computational process.

The RTS smoother consists of a common forward Kalman filter and a backward smoother. The backward sweep begins at the end of the forward Kalman filter. Figure 4 illustrates the computation procedure of the RTS smoother.

The RTS algorithm can be expressed in discrete form as shown by Equation (28) [17,18,20]:

(28) $$\begin{array}{l} H_{k}=P_{fk}^{+}\Phi_{k}^{T}{\left(P_{fk+1}^{-}\right)}^{-1}\\ {\hat{x}}_{k}={\hat{x}}_{fk}^{+}+H_{k}\left[{\hat{x}}_{k+1}-{\hat{x}}_{fk+1}^{-}\right]\\ P_{k}=P_{fk}^{+}-H_{k}\left[P_{fk+1}^{-}-P_{k+1}\right]H_{k}^{T} \end{array}$$

where $H_{k}$ is the smoothing gain matrix. $\Phi_{k}$ is the system state transition matrix. ${\hat{x}}_{fk+1}^{-}$ is the optimal predicted estimate at epoch k+1 and $P_{fk+1}^{-}$ represents its covariance matrix.

The RTS algorithm is the easiest and simplest smoothing method in implementation [18]. Here the RTS smoother will be used for estimating the states in the surveying intervals.

## 5. Surveying Accuracy Analysis of the New Algorithm

The surveying accuracy of the proposed algorithm can be presented by the error state covariance matrix, which can be obtained by calculating analytically the Riccati equation of the system. The analysis shows that it is difficult to obtain the accurate analytical solution of RTS smoothing algorithm, but fortunately, it has been demonstrated that the TFS and RTS smoother are mathematically equivalent in linear cases [15,17,18,19,20]. We can analyze the surveying accuracy by calculating the state covariance matrix of the equivalent TFS.

According to Equation (27), the covariance matrixes of the forward filter and the backward filter need to be calculated. For the vertical channel, the Riccati equation in continuous form of forward filter is [18]:

(29) $$\frac{d}{dt}P_{vf}\left(t\right)=A_{v}P_{vf}\left(t\right)+P_{vf}\left(t\right)A_{v}^{T}-P_{vf}\left(t\right)H_{v}^{T}R_{v}^{-1}H_{v}P_{vf}\left(t\right)+G_{v}Q_{v}G_{v}^{T}$$

For the surveying process of every interval, the observation of position is measured at the end time epoch T. Consider that $P_{v}\left(T\right)=P_{vf}\left(T\right)$, the solution of the Riccati equation at other time epoch can be expressed as shown in Equation (30) [18]:

(30) $$P_{vf}\left(t\right)=\Phi_{v}\left(t,0\right)P_{vf}\left(0\right)\Phi_{v}^{T}\left(t,0\right)+\int_{0}^{t}\Phi_{v}\left(t-\tau \right)G_{v}Q_{v}G_{v}^{T}\Phi_{v}^{T}\left(t-\tau \right)d\tau$$

where the system state transition matrix is:

(31) $$\Phi_{v}\left(t,0\right)=e^{A_{v}t}=\left[\begin{array}{ccc} 1 & v_{odo}t & -\frac{1}{2}v_{odo}t^{2}\\ 0 & 1 & -t\\ 0 & 0 & 1 \end{array}\right]$$

Let the initial covariance matrix be:

(32) $$P_{vf}\left(0\right)=diag\left(\left[\begin{array}{ccc} p_{r_{D}} & p_{{\bar{\phi }}_{pitch}} & p_{{\bar{\omega }}_{y}} \end{array}\right]\right)$$

Substituting $G_{v}$, $Q_{v}$and Equations (31) and (32) into Equation (30) and rearranging we can obtain the covariance matrix of forward filter as shown by Equation (33):

(33) $$P_{vf}\left(t\right)=\left[\begin{array}{ccc} p_{r_{D}}+p_{{\bar{\phi }}_{pitch}}v_{odo}^{2}t^{2}+\frac{1}{4}p_{{\bar{\omega }}_{y}}v_{odo}^{2}t^{4}+\frac{1}{3}\sigma_{w_{pitch}}^{2}v_{odo}^{2}t^{3} & p_{{\bar{\phi }}_{pitch}}v_{odo}t+\frac{1}{2}p_{{\bar{\omega }}_{y}}v_{odo}t^{3}+\frac{1}{2}\sigma_{w_{pitch}}^{2}v_{odo}t^{2} & -\frac{1}{2}p_{{\bar{\omega }}_{y}}v_{odo}t^{2}\\ p_{{\bar{\phi }}_{pitch}}v_{odo}t+\frac{1}{2}p_{{\bar{\omega }}_{y}}v_{odo}t^{3}+\frac{1}{2}\sigma_{w_{pitch}}^{2}v_{odo}t^{2} & p_{{\bar{\phi }}_{pitch}}+p_{{\bar{\omega }}_{y}}t^{2}+\sigma_{w_{pitch}}^{2}t & -p_{{\bar{\omega }}_{y}}t\\ -\frac{1}{2}p_{{\bar{\omega }}_{y}}v_{odo}t^{2} & -p_{{\bar{\omega }}_{y}}t & p_{{\bar{\omega }}_{y}} \end{array}\right]$$

The Riccati equation in continuous form of backward filter is [18]:

(34) $$\frac{d}{dt}P_{vb}\left(t\right)=A_{v}P_{vb}\left(t\right)+P_{vb}\left(t\right)A_{v}^{T}-G_{v}Q_{v}G_{v}^{T}+P_{vb}\left(t\right)H_{v}^{T}R_{v}^{-1}H_{v}P_{vb}\left(t\right)$$

This equation should be integrated inversely in time (T→t) with the initial conditions of ${\left(P_{vb}^{-}\left(T\right)\right)}^{-1}={\left(P_{vbN}^{-}\right)}^{-1}=0$ [18]. Since observation of position for the backward filter only be provided at the initial time epoch T, we can use the discrete Kalman filter equation to calculate the covariance matrix of the updated estimate of error state at epoch T for the backward filter. We can thus obtain:

(35) $${\left(P_{vb}^{+}\left(T\right)\right)}^{-1}={\left(P_{vbN}^{+}\right)}^{-1}={\left(P_{vbN}^{-}\right)}^{-1}+H_{vN}^{T}R_{vN}^{-1}H_{vN}=H_{vN}^{T}R_{vN}^{-1}H_{vN}=\left[\begin{array}{ccc} 0 & 0 & 0\\ 0 & \frac{1}{\sigma_{r}^{2}} & 0\\ 0 & 0 & 0 \end{array}\right]$$

The solution of the backward filter Riccati equation at other time epoch can be expressed by Equation (36):

(36) $$P_{vb}\left(t\right)=\Phi_{v}\left(t,T\right)P_{vb}^{+}\left(T\right)\Phi_{v}^{T}\left(t,T\right)-\int_{T}^{t}\Phi_{v}\left(t,\tau \right)G_{v}Q_{v}G_{v}^{T}\Phi_{v}^{T}\left(t,\tau \right)d\tau$$

Considering that ${\left(P_{vb}^{+}\left(T\right)\right)}^{-1}$ is a singular matrix, we can use the Sherman-Morrison-Woodbury formula [18] to calculate $P_{vb}^{-1}\left(t\right)$. The formula can be expressed by Equation (37):

(37) $${\left(F+BCD\right)}^{-1}=F^{-1}-F^{-1}B{\left(DF^{-1}B+C^{-1}\right)}^{-1}DF^{-1}$$

Defining $F=\Phi_{v}\left(t,T\right)P_{vb}\left(T\right)\Phi_{v}^{T}\left(t,T\right),B=C=I,D=-\int_{T}^{t}\Phi_{v}\left(t,\tau \right)G_{v}Q_{v}G_{v}^{T}\Phi_{v}^{T}\left(t,\tau \right)d\tau$. Inverting Equation (36) we have:

(38) $$\begin{array}{ll} P_{vb}^{-1}\left(t\right) & ={\left[\Phi_{v}\left(t,T\right)P_{vb}\left(T\right)\Phi_{v}^{T}\left(t,T\right)-\int_{T}^{t}\Phi_{v}\left(t,\tau \right)G_{v}Q_{v}G_{v}^{T}\Phi_{v}^{T}\left(t,\tau \right)d\tau \right]}^{-1}\\ & =\Phi_{v}^{T}\left(T,t\right)P_{vb}^{-1}\left(T\right)\Phi_{v}\left(T,t\right)-\Phi_{v}^{T}\left(T,t\right)P_{vb}^{-1}\left(T\right)\Phi_{v}\left(T,t\right)\\ & {\left(-\left[\int_{T}^{t}\Phi_{v}\left(t,\tau \right)G_{v}Q_{v}G_{v}^{T}\Phi_{v}^{T}\left(t,\tau \right)d\tau \right]\Phi_{v}^{T}\left(T,t\right)P_{vb}^{-1}\left(T\right)\Phi_{v}\left(T,t\right)+I\right)}^{-1}\\ & \left[-\int_{T}^{t}\Phi_{v}\left(t,\tau \right)G_{v}Q_{v}G_{v}^{T}\Phi_{v}^{T}\left(t,\tau \right)d\tau \right]\Phi_{v}^{T}\left(T,t\right)P_{vb}^{-1}\left(T\right)\Phi_{v}\left(T,t\right) \end{array}$$

where $\Phi_{v}\left(T,t\right)=e^{A_{v}\left(T-t\right)}$. Substituting ${\left(P_{vb}^{+}\left(T\right)\right)}^{-1}$, $G_{v}$ and $Q_{v}$ into Equation (38) yields:

(39) $$P_{vb}^{-1}\left(t\right)=\frac{1}{12\sigma_{r}^{2}-4v_{odo}^{2}{\left(t-T\right)}^{3}\sigma_{w_{pitch}}^{2}}\left[\begin{array}{ccc} 12 & 12v_{odo}\left(t-T\right) & -6v_{odo}{\left(t-T\right)}^{2}\\ 12v_{odo}\left(t-T\right) & 12v_{odo}^{2}{\left(t-T\right)}^{2} & 6v_{odo}^{2}{\left(t-T\right)}^{3}\\ -6v_{odo}{\left(t-T\right)}^{2} & 6v_{odo}^{2}{\left(t-T\right)}^{3} & 3v_{odo}^{2}{\left(t-T\right)}^{4} \end{array}\right]$$

Substituting Equations (33) and (39) into Equation (27), the covariance of the optimal smoothed estimate of vertical position error can be obtained as indicated in Equation (40):

(40) $$\begin{array}{ll} p_{r_{D}}\left(t\right) & =p_{r_{D}}-\frac{p_{r_{D}}\left(12p_{r_{D}}+12\sigma_{r}^{2}+12p_{{\bar{\phi }}_{pitch}}v_{odo}^{2}\left(2tT-t^{2}\right)+4\sigma_{w_{pitch}}^{2}v_{odo}^{2}\left(3t^{2}T-2t^{3}\right)+3p_{{\bar{\omega }}_{y}}v_{odo}^{2}\left(2t^{2}T^{2}-t^{4}\right)\right)}{12p_{r_{D}}+12\sigma_{r}^{2}+12p_{{\bar{\phi }}_{pitch}}v_{odo}^{2}T^{2}+4\sigma_{w_{pitch}}^{2}v_{odo}^{2}T^{3}+3p_{{\bar{\omega }}_{y}}v_{odo}^{2}T^{4}}\\ & +\frac{\sigma_{r}^{2}\left(12p_{r_{D}}+12\sigma_{r}^{2}+12p_{{\bar{\phi }}_{pitch}}v_{odo}^{2}t^{2}+4\sigma_{w_{pitch}}^{2}v_{odo}^{2}t^{3}+3p_{{\bar{\omega }}_{y}}v_{odo}^{2}t^{4}\right)-12\sigma_{r}^{4}}{12p_{r_{D}}+12\sigma_{r}^{2}+12p_{{\bar{\phi }}_{pitch}}v_{odo}^{2}T^{2}+4\sigma_{w_{pitch}}^{2}v_{odo}^{2}T^{3}+3p_{{\bar{\omega }}_{y}}v_{odo}^{2}T^{4}}\\ & -\frac{v_{odo}^{4}t^{2}{\left(t-T\right)}^{2}\left(\sigma_{w_{pitch}}^{4}t\left(t-4T\right)+9p_{{\bar{\phi }}_{pitch}}p_{{\bar{\omega }}_{y}}T^{2}+3\sigma_{w_{pitch}}^{2}T\left(4p_{{\bar{\phi }}_{pitch}}+p_{{\bar{\omega }}_{y}}tT\right)\right)}{3\left(12p_{r_{D}}+12\sigma_{r}^{2}+12p_{{\bar{\phi }}_{pitch}}v_{odo}^{2}T^{2}+4\sigma_{w_{pitch}}^{2}v_{odo}^{2}T^{3}+3p_{{\bar{\omega }}_{y}}v_{odo}^{2}T^{4}\right)} \end{array}$$

where $p_{r_{D}}$ is the initial positon error variance. $\sigma_{r}^{2}$ is the PSD of position measurement noise. $p_{{\bar{\phi }}_{pitch}}v_{odo}^{2}T^{2}$, $\sigma_{w_{pitch}}^{2}v_{odo}^{2}T^{3}$ and $p_{{\bar{\omega }}_{y}}v_{odo}^{2}T^{4}$ represent the vertical position error variance caused by the initial pitch error, the equivalent random walk noise and bias instability of the pitch axis, respectively.

For the level channel, the system state transition matrix and the initial covariance matrix can be expressed using Equation (41):

(41) $$\Phi_{l}\left(t,0\right)=e^{A_{l}t}=\left[\begin{array}{ccccc} 1 & 0 & v_{odo}t\cos\varphi & v_{odo}t\sin\varphi & -\frac{1}{2}v_{odo}t^{2}\sin\varphi \\ 0 & 1 & v_{odo}t\sin\varphi & -v_{odo}t\cos\varphi & \frac{1}{2}v_{odo}t^{2}\cos\varphi \\ 0 & 0 & 1 & 0 & 0\\ 0 & 0 & 0 & 1 & -t\\ 0 & 0 & 0 & 0 & 1 \end{array}\right]\;P_{lf}\left(0\right)=diag\left(\left[\begin{array}{ccccc} p_{r} & p_{r} & p_{\delta k} & p_{{\bar{\phi }}_{D}} & p_{{\bar{\omega }}_{z}} \end{array}\right]\right)$$

We can calculate the covariance of the smoothed estimate of north and east position error in the same way as shown in Equations (34)–(38). The solutions are very complex, but can be written as the following Equation (42) shows:

(42) $$\begin{array}{l} p_{r_{N}}\left(t\right)=p_{0}\left(t\right)-p_{1}\left(t\right)\cos\left(2\varphi \right)\\ p_{r_{E}}\left(t\right)=p_{0}\left(t\right)+p_{1}\left(t\right)\cos\left(2\varphi \right) \end{array}\;\left(p_{0}\left(t\right)>0,\;p_{1}\left(t\right)>0\right)$$

where φ is the yaw angle of the trolley with respect to the navigation frame. Considering that −1≤cos(2φ)≤1, the variation range of $p_{r_{N}}\left(t\right)$ and $p_{r_{E}}\left(t\right)$ is $\left[p_{0}\left(t\right)-p_{1}\left(t\right),p_{0}\left(t\right)+p_{1}\left(t\right)\right]$. In fact, $p_{0}\left(t\right)-p_{1}\left(t\right)$ represents the variance of longitudinal position error, and $p_{0}\left(t\right)+p_{1}\left(t\right)$ represents the variance of lateral position error. In order to facilitate analysis, we can project the north and east position error onto the longitudinal (forward) and lateral (right) directions by the projection formula as shown by Equation (43):

(43) $$\left[\begin{array}{cc} p_{L}\left(t\right) & p_{LR}\left(t\right)\\ p_{LR}\left(t\right) & p_{R}\left(t\right) \end{array}\right]=\left[\begin{array}{cc} \cos\varphi & \sin\varphi \\ -\sin\varphi & \cos\varphi \end{array}\right]\left[\begin{array}{cc} p_{r_{N}}\left(t\right) & p_{r_{NE}}\left(t\right)\\ p_{r_{NE}}\left(t\right) & p_{r_{E}}\left(t\right) \end{array}\right]\left[\begin{array}{cc} \cos\varphi & -\sin\varphi \\ \sin\varphi & \cos\varphi \end{array}\right]$$

The analytical solution of the longitudinal position error variance is given by Equation (44):

(44) $$\begin{array}{ll} p_{L}\left(t\right) & =p_{r}-\frac{p_{r}\left(3p_{r}+3\sigma_{r}^{2}+3p_{\delta k}v_{odo}^{2}\left(2tT-t^{2}\right)+\sigma_{w_{\delta k}}^{2}v_{odo}^{2}\left(3t^{2}T-2t^{3}\right)\right)}{3p_{r}+3\sigma_{r}^{2}+3p_{\delta k}v_{odo}^{2}T^{2}+\sigma_{w_{\delta k}}^{2}v_{odo}^{2}T^{3}}\\ & +\frac{\sigma_{r}^{2}\left(3p_{r}+3\sigma_{r}^{2}+3p_{\delta k}v_{odo}^{2}t^{2}+v_{odo}^{2}\sigma_{w_{\delta k}}^{2}t^{3}\right)-3\sigma_{r}^{4}}{3p_{r}+3\sigma_{r}^{2}+3p_{\delta k}v_{odo}^{2}T^{2}+\sigma_{w_{\delta k}}^{2}v_{odo}^{2}T^{3}}\\ & -\frac{\sigma_{w_{\delta k}}^{2}v_{odo}^{4}t^{2}{\left(t-T\right)}^{2}\left(\sigma_{w_{\delta k}}^{2}t\left(t-4T\right)-12p_{\delta k}T\right)}{12\left(3p_{r}+3\sigma_{r}^{2}+3p_{\delta k}v_{odo}^{2}T^{2}+\sigma_{w_{\delta k}}^{2}v_{odo}^{2}T^{3}\right)} \end{array}$$

where $p_{\delta k}v_{odo}^{2}T^{2}$ represents the longitudinal position error variance caused by the initial variance of scale factor error. And $\sigma_{w_{\delta k}}^{2}v_{odo}^{2}T^{3}$ represents the longitudinal position error variance caused by the random noise of scale factor error. The lateral position error variance is:

(45) $$\begin{array}{ll} p_{R}\left(t\right)= & p_{r}-\frac{p_{r}\left(12p_{r}+12\sigma_{r}^{2}+12p_{{\bar{\phi }}_{D}}v_{odo}^{2}\left(2tT-t^{2}\right)+4\sigma_{w_{D}}^{2}v_{odo}^{2}\left(3t^{2}T-2t^{3}\right)+3p_{{\bar{\omega }}_{z}}v_{odo}^{2}\left(2t^{2}T^{2}-t^{4}\right)\right)}{12p_{r}+12\sigma_{r}^{2}+12p_{{\bar{\phi }}_{D}}v_{odo}^{2}T^{2}+4\sigma_{w_{D}}^{2}v_{odo}^{2}T^{3}+3p_{{\bar{\omega }}_{z}}v_{odo}^{2}T^{4}}\\ & +\frac{\sigma_{r}^{2}\left(12p_{r}+12\sigma_{r}^{2}+12p_{{\bar{\phi }}_{D}}v_{odo}^{2}t^{2}+4\sigma_{w_{D}}^{2}v_{odo}^{2}t^{3}+3p_{{\bar{\omega }}_{z}}v_{odo}^{2}t^{4}\right)-12\sigma_{r}^{4}}{12p_{r}+12\sigma_{r}^{2}+12p_{{\bar{\phi }}_{D}}v_{odo}^{2}T^{2}+4\sigma_{w_{D}}^{2}v_{odo}^{2}T^{3}+3p_{{\bar{\omega }}_{z}}v_{odo}^{2}T^{4}}\\ & -\frac{v_{odo}^{4}t^{2}{\left(t-T\right)}^{2}\left(\sigma_{w_{D}}^{4}t\left(t-4T\right)+9p_{{\bar{\phi }}_{D}}p_{{\bar{\omega }}_{z}}T^{2}+3\sigma_{w_{D}}^{2}T\left(4p_{{\bar{\phi }}_{D}}+p_{{\bar{\omega }}_{z}}tT\right)\right)}{3\left(12p_{r}+12\sigma_{r}^{2}+12p_{{\bar{\phi }}_{D}}v_{odo}^{2}T^{2}+4\sigma_{w_{D}}^{2}v_{odo}^{2}T^{3}+3p_{{\bar{\omega }}_{z}}v_{odo}^{2}T^{4}\right)} \end{array}$$

where $p_{{\bar{\phi }}_{D}}v_{odo}^{2}T^{2}$, $\sigma_{w_{D}}^{2}v_{odo}^{2}T^{3}$ and $p_{{\bar{\omega }}_{z}}v_{odo}^{2}T^{4}$ represent the lateral position error variance caused by the initial yaw error, the equivalent random walk noise and bias instability of the yaw axis, respectively.

As shown in Equations (40), (44) and (45), the vertical position error is mainly affected by the lateral direction gyro drifts and initial pitch error, the lateral position error by the vertical direction gyro drifts and initial orientation error, and the longitudinal position error is affected by the odometer.

In order to verify the correctness of the derived analytical solution of the covariance matrix, a comparison with the numerical solution should be performed. Setting *T* = 60 s, *v_odo_* = 1 m/s, and the initial covariance matrix, spectral density matrix of system noise and measurement noise covariance matrix are set as indicated in Equation (46):

(46) $$\begin{array}{l} P_{v}\left(0\right)=diag\left(\left[\begin{array}{ccc} {\left(0.6\;mm\right)}^{2} & {\left(0.005\;{}^{\circ}\right)}^{2} & {\left(0.05\;{}^{\circ}/h\right)}^{2} \end{array}\right]\right)\\ Q_{v}={\left(0.02\;{}^{\circ}/\sqrt{h}\right)}^{2}\\ R_{v}={\left(0.6\;mm\right)}^{2}\\ P_{l}\left(0\right)=diag\left(\left[\begin{array}{ccccc} {\left(0.6\;mm\right)}^{2} & {\left(0.6\;mm\right)}^{2} & {\left(0.002\right)}^{2} & {\left(0.2\;{}^{\circ}\right)}^{2} & {\left(0.05\;{}^{\circ}/h\right)}^{2} \end{array}\right]\right)\\ Q_{l}=diag\left(\left[\begin{array}{cc} {\left(0.000008/\sqrt{s}\right)}^{2} & {\left(0.02\;{}^{\circ}/\sqrt{h}\right)}^{2} \end{array}\right]\right)\\ R_{l}=diag\left(\left[\begin{array}{cc} {\left(0.6\;mm\right)}^{2} & {\left(0.6\;mm\right)}^{2} \end{array}\right]\right) \end{array}$$

**Figure 5:**
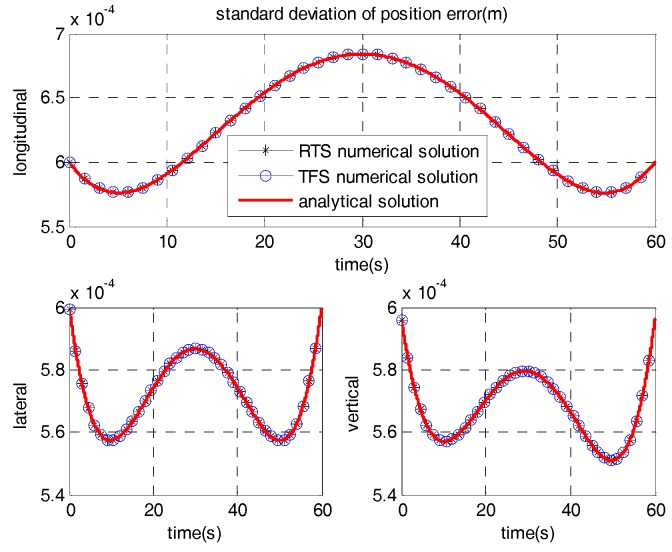
Comparison of RTS and TFS numerical solutions with the analytical solution.

The standard deviations of position errors calculated by the analytical solution and numerical solution of the RTS algorithm and TFS algorithm are shown in Figure 5. As Figure 5 illustrates, the analytical solution of the covariance matrix is identical with numerical solution and the smoothing effect of RTS and TFS are equivalent.

We can take advantage of the analytical solution of the covariance matrix to quantitatively analyze the position errors introduced by gyro drifts and odometer errors for guiding the selection of the sensors. For this purpose, the variances of initial attitude angle errors, initial position errors and measurement noise need to be set to typical values. According to the precision of the total station, we set $p_{r}=\sigma_{r}^{2}={\left(0.6\;\mathrm{mm}\right)}^{2}$. Considering that the accelerometer used in the system with a bias of 50 μg will give rise to a level error of about 0.003° [16], and equivalent level error contains the residual misalignment error between the IMU and odometer, here we set $p_{{\bar{\phi }}_{pitch}}={\left(0.005{}^{\circ}\right)}^{2}$. The initial orientation error is approximately determined by the gyro bias and the local latitude, that is $\phi_{D}=\delta \omega_{g}/\left(\omega_{ie}\cos L\right)$. In addition, the surveying distance of each interval is 60 m and the velocity is set to 1 m/s. The observation of position is provided only at the initial and the end of time epoch, the maximum of smoothed position error caused by gyro bias instability and random walk noise exists in the middle of surveying time and setting *t* = *T*/2.

**Figure 6:**
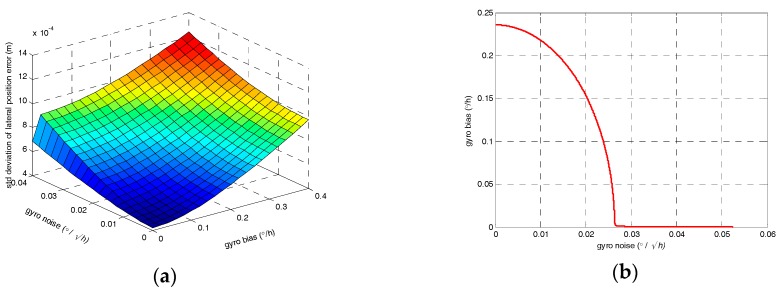

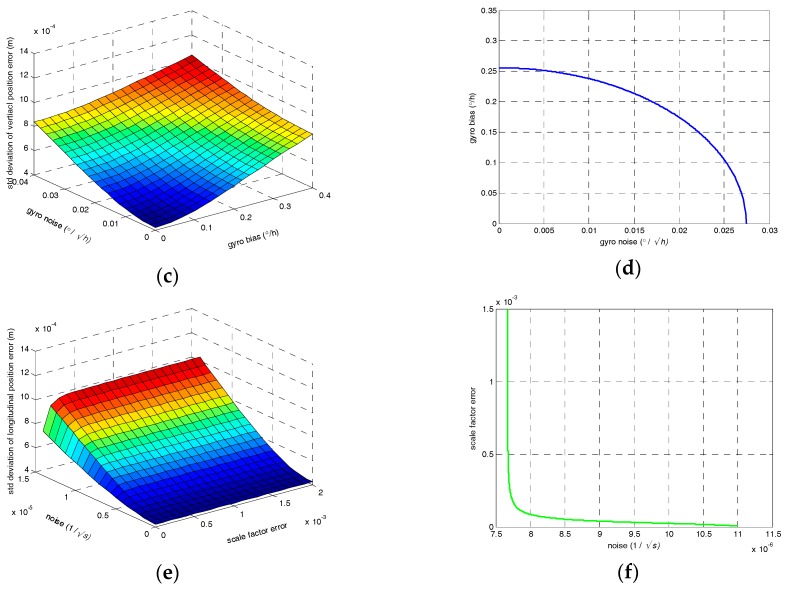
(**a**) Theoretical standard deviation of lateral position error; (**b**) The relationship of drifts for the equivalent vertical gyro; (**c**) Theoretical standard deviation of vertical position error; (**d**) The relationship of drifts for the equivalent lateral gyro; (**e**) Theoretical standard deviation of longitudinal position error; (**f**) The relationship of drifts for the odometer.

Figure 6 illustrates the relationship of vertical and lateral position error standard deviation with the gyro bias instability and random walk noise theoretically. As Figure 6b,d,f illustrate, in order to satisfy the surveying accuracy of 0.67 mm (1 σ) for the longitudinal direction, the uncertainty of odometer scale factor error should less than 0.000011 /$\sqrt{s}$ at most. The scale factor is a function of position, and its uncertainty is caused by the change of position. The velocity has a certain value so that the uncertainty of the scale factor becomes a function of time. For the lateral and vertical directions, the values of equivalent gyro bias and equivalent random walk noise should below the lines in Figure 6b,d.

In fact, the equivalent gyro drifts contain the terms caused by the projections of the Earth’s and azimuth’s rotation rate through the attitude errors as expressed in Equation (6). The constant part of the attitude errors results in the equivalent constant gyro bias. The drift part of the attitude errors results in the drift of the equivalent gyro bias. Since the attitude error changes slowly, the drift part can be ignored for a short period of time measurement.

For the RLG used in our surveying system, the gyro bias instability is about 0.03°/h which will result in an orientation alignment error of about 0.18° [16]. According to Equation (6), the estimated value of the incremental equivalent gyro bias introduced by the projection of the Earth’s and azimuth’s rotation rate is about 0.05°/h at most. Considering that its random walk noise is about 0.005°/$\sqrt{h}$, the gyros used in the system can satisfy the demands of the surveying accuracy.

For a typical Fiber Optic Gyro (FOG) with bias instability of 0.1°/h and random walk noise of 0.007°/$\sqrt{h}$, the orientation error is about 0.6° and the equivalent gyro bias is about 0.25°/h, which exceeds the requirement according to Figure 6 and therefore, it cannot satisfy the surveying accuracy demand of 0.67 mm (1 σ).

In addition, the theoretical analysis result is obtained under ideal conditions and only the bias instability and the random walk noise of gyros are considered. The actual noise models of gyros are more complex than that. We should select gyroscopes with better performance than the index as Figure 6 shows in practice and the rate random walk and Markov process noise of the gyros should also be small.

## 6. Simulations and Experimental Results

### 6.1. Simulations

**Figure 7:**
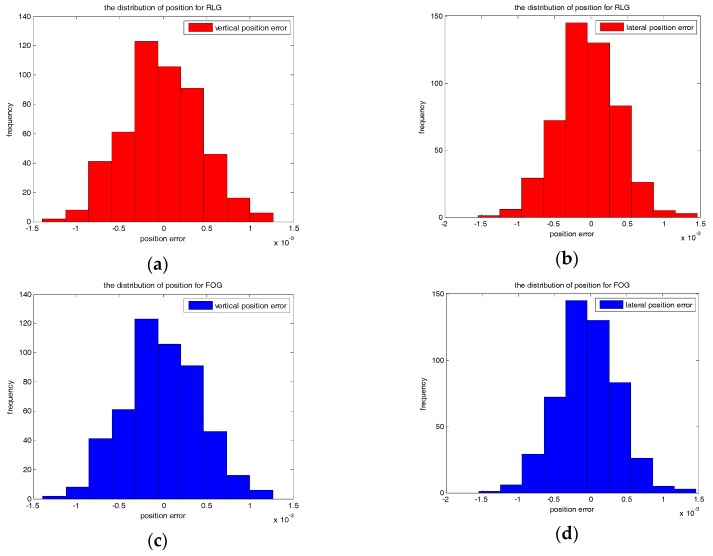
(**a**) The distribution of vertical position error for the RLG-based IMU; (**b**) The distribution of lateral position error for the RLG-based IMU; (**c**) The distribution of vertical position error for the FOG-based IMU; (**d**) The distribution of vertical position error for the FOG-based IMU.

Firstly, Monte Carlo simulations of the surveying accuracy for the proposed algorithm have been implemented based on the real random noises of two different grade gyroscopes. The simulated trajectory is a straight line and $C_{b}^{n}=I$. The random noises are measured by a RLG-based IMU and a FOG-based IMU in static state. For the RLG-based IMU, the PSD of random walk noise is about 0.005°/$\sqrt{h}$, and the bias instability is set to 0.03°/h, the variance of initial orientation error is set to (0.2°)^2^. For the FOG based IMU, the PSD of random walk noise is about 0.007°/$\sqrt{h}$, and the bias instability is set to 0.1°/h, the variance of initial orientation error is set to 0.6°. Commonly the variance of initial level error is set to (0.005°)^2^. The variances of the initial position error and the position observation noise are set to (0.6 mm)^2^. We take the position error at middle of the time to test the statistical accuracy. Five hundred groups of Monte Carlo simulation results of vertical and lateral directions are shown in Figure 7.

According to Figure 7, the Root Mean Square (RMS) of the surveying accuracy for the RLG-based IMU is about 0.44 mm (1σ) for the vertical direction and 0.42 mm (1σ) for the lateral direction. The RMS of the surveying accuracy for the FOG-based IMU is about 1.71 mm (1σ) for the vertical direction and 0.77 mm (1σ) for the lateral direction. This is consistent to the result calculated by the covariance analysis.

**Figure 8:**
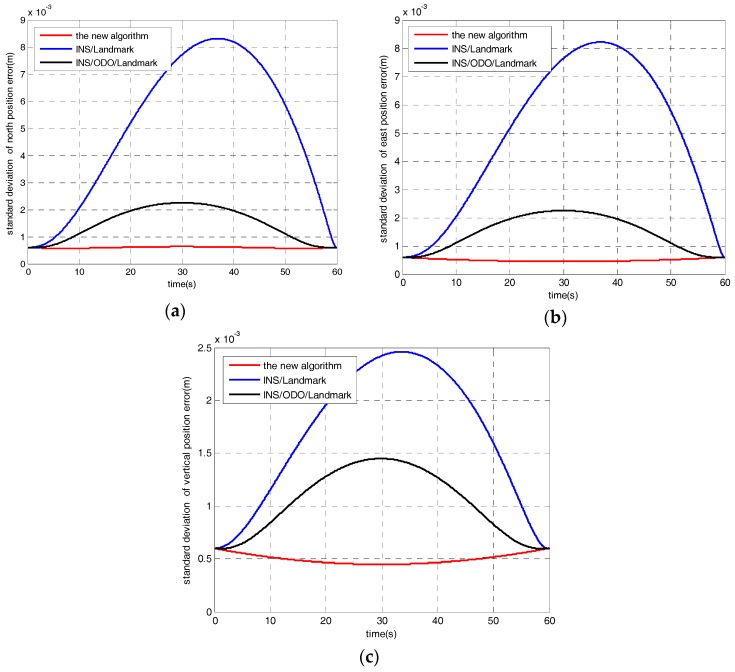
(**a**) Accuracy comparison with different algorithms in the north direction; (**b**) Accuracy comparison with different algorithms in the east direction; (**c**) Accuracy comparison with different algorithms in the vertical direction.

Secondly, surveying accuracy simulations for the proposed algorithm compared with traditional filtering algorithms, namely INS/landmark integration and INS/odometer/landmark integration, have been carried out based on the numerical analysis of covariance. The gyro drifts are set to 0.01°/h of bias instability and 0.005°/$\sqrt{h}$ of random walk noise. The accelerometer drifts are set to 10 μg of bias instability and 5 $\mu g/\sqrt{\mathrm{Hz}}$ of random noise. Setting *T* = 60 s, *v* = 1 m/s, and position observations are provided at the end of the time. The RTS smoothers are employed when the process of forward filtering is finished for all algorithms. The simulation results are shown in Figure 8, which illustrates, that the surveying accuracy of the proposed algorithm is better than that of the traditional filtering algorithms based on INS or INS/odometer integrations. The bias of the inertial sensors cannot be estimated well under railway surveying applications by the traditional algorithms, which need inertial sensors of higher precision grade for long distance interval surveying or more high accuracy position updates.

For the new algorithm of this paper, the equivalent gyro biases can be estimated and compensated. Therefore, the proposed algorithm is a low-cost and high-efficiency approach with respect to the traditional ones.

### 6.2. Experimental Results

**Figure 9:**
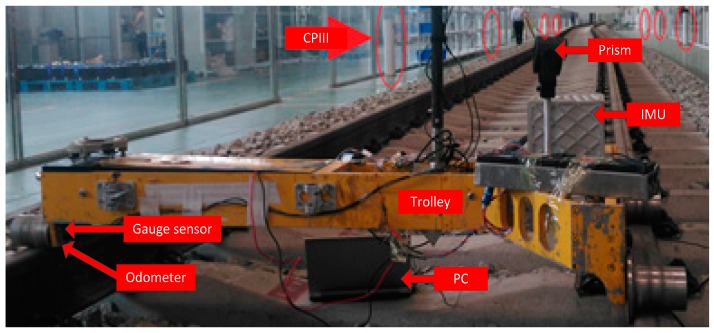
The experimental railway track surveying system.

Real tests were carried out on the experimental railway line of the Zhu Zhou Time Electronic Technology Company (Hunan, China). The test length of the track is 120 m as illustrated in Figure 9. The absolute position is measured by a Leica high precision total station with a prism mounted on the trolley based on the CPIII control points shown in the figure.

At the beginning of the experiments we put the surveying trolley on the starting position of the track for 15 min of static initial alignment. The initial position is measured by the total station. Then pushing the trolley moves forward it on the track at walking speed providing the observations of absolute position every 60 m in the stationary state. Six groups of 120 m long track surveying experiments have been carried out. The system was repowered and realigned after every three groups of experiments.

**Figure 10:**
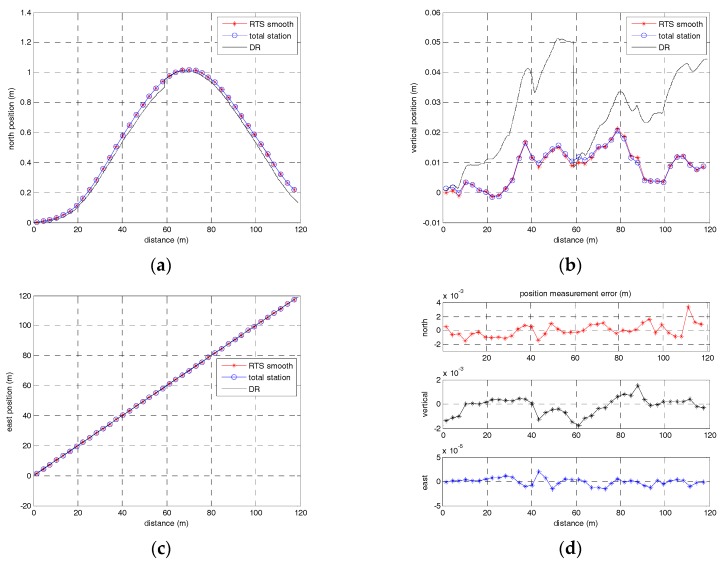
(**a**) The north position measurements; (**b**) The vertical position measurements; (**c**) The east position measurements; (**d**) The position measurement errors of north, east and vertical directions.

The true values of absolute positions about the railway line are measured by the high total station (0.6 mm, 0.5”) in static mode based on the CPIII control network. We compare the smoothed surveying results with the absolute position measured by the total station in every 3 m interval, one of which was illustrated in Figure 10. The RMS of position errors of the six groups of surveying results was calculated, respectively, and is listed in Table 1.

As shown in Figure 10 and Table 1, the absolute positions identified by the approach of the new filtering and smoothing algorithm are in good agreement with those measured by the traditional total station approach in static mode. The new algorithm proposed in this paper is effective, and the absolute position measuring accuracy can reach 1 mm (1σ) with position observation measured by a total station every 60 m interval for the real tests. Therefore, the approach based on the new algorithm can satisfy the demands of high absolute accuracy and works efficiently for railway track surveying compared with the traditional total station approach.

## 7. Conclusions

Railway track surveying is an essential step in the process of railway construction and maintenance. In order to overcome the shortcomings of traditional track surveying approaches, this paper proposes a new filtering and smoothing algorithm based on IMU/odometer data and landmarks for high precise railway track surveying.

The approach in this paper takes use of the IMU/odometer based dead reckoning system integrated with landmarks measured by a total station for high precise track surveying. A new model with completely observable error states for the Kalman filter and smoother is established by combining error terms to overcome the difficulty of estimating too many error parameters with too few observations of landmarks under weak maneuver conditions.

Since the new filtering and smoothing algorithm can estimate and compensate the equivalent gyro biases of the system, it can reduce the accuracy requirement of the gyros and the frequency requirement of the position observations. Therefore the surveying approach based on the new algorithm is effective in reducing the cost and improving work efficiency compared with traditional integration algorithms based on INS.

Analytical solutions of position error variance for longitudinal, lateral and vertical directions were presented by solving the covariance propagation equation of the new filtering and smoothing algorithm, which can be used in analyzing the accuracy of measurement result in theory. It is significant for guiding the selection of the gyroscopes. According to the covariance analysis and simulation results, gyros with bias instability under 0.1°/h and random walk noise less than 0.005°$\sqrt{h}$ at the same time can satisfy the surveying accuracy requirements. Due to only the bias instability and the random walk noise are considered in the model, the rate random walk and Markov noise should also be small for the gyros.

Experimental results illustrated that the absolute accuracy of the new approach with respect to the measurement of total station in static mode for railway track surveying can reach 1 mm (1σ) when using RLG based IMU with gyro bias instability of 0.03°/h and random walk noise of 0.005°/$\sqrt{h}$. The interval of position observation based on total station can be extended to 60 m in length, which can reduce the measuring time significantly. The approach can simultaneously satisfy the demands of high accuracy and work efficiency for railway track surveying.

## Figures and Tables

**Table 1:** RMS values (1σ) of the new filtering and smoothing algorithm for 6 groups of railway track surveying experiments.

RMS (mm)	Group 1	Group 2	Group 3	Group 4	Group 5	Group 6
North	1.0938	1.2332	0.9374	0.9890	0.8838	0.8902
Vertical	0.9118	0.9285	0.7052	0.7735	0.7981	0.9148
East	0.0090	0.0096	0.0077	0.0088	0.0084	0.0087
